# Indents

**Published:** 1911-12-15

**Authors:** 


### INDENTS.

**☞Brief Articles of Technical or Scientific Value will receive notice and comment. Contributions invited.**

*Some Untoward Facts Regarding Fletcherism.* Perhaps no department of medicine, in practice, ignores so completely the old established principles of biology and physiology as dietetics. Albeit, it is well known that digestion does not entail the proper absorption, and hence the assimilation, which prevents intestinal toxemia; while it is definitely settled that the human body is wrapped around a tube, and what is in the tube is not yet in the body; while it is also established that starch and sugar digestion takes place partly in the mouth, protein chiefly in the stomach and partly in the intestines, while starch, sugar and fat are digested chiefly in the intestines; still, indigestion is regarded as of mouth or stomach origin, hence the popularity of mastication and pepsin nostrums for dyspepsia, irrespective of its character.

Furthermore, digestion, which is the preparation of food for absorption and assimilation, is regarded as the end and not as the means. All sorts of morbid conditions charged to indigestion are often due to fermantative changes in food that has been properly digested in too great quantities for an organism unsuited for its absorption and assimilation. Although many states, like scurvy in children, hepatic and renal instabilities in the insane and neuropaths, result from monotany in diet, and while many blood-vessel degenerations and strains result from hyperacidoses and toxemias produced by undue use of carbohydrates, still the old myth that all these states are due to an undue meat diet serenely dominates dietetics, despite all physiologico-chemic revelations to the contrary.

One popular fad which ignores these physiologic axioms is Fletcherism. The proper mastication of food is prescribed as the nostrum for all assimilative and absorptive digestion disorders. One natural result of this fad is the excessive or disproportionate use of starchy and sugary foods. These substances, as has already been pointed out in *The American Journal of Clinical Medicine*, are very frequent sources of paralyses, occurring in persons who do not eat meat excessively and who abstain from alcohol.

The rationale of such paralysis is simple. When people enter on the climacteric, between forty-five and sixty, there is a potentiality of blood-vessel changes, and a general upset of the constitution from diminution cf function. If there be no excessive strain at this time the new physiologic balance is attained and the organism resumes its usual health. A mental or physical strain at this time is apt to effect the blood vessels and elimination through its influence on the liver and kidneys. If diet be adjusted to elimination no strain results, but seeming facility of digestion, as the supposed result of thorough mastication, is apt to lead to hemic hyperacidity, usually resulting through the preponderance of carbohydrate food, which lends itself readily to fermentative changes and hyperacidity; and, also, this dietetic habit results in an indicanemia, through the morbid ready absorption of waste protein. These absorbed materials are great sources of blood-vessel strain.

As reliance on Fletcherism alone produces not only these, but a tendency to monotony of allegedly easily digested foods the untoward effects of Fletcherism are liable to be peculiarly dangerous at the periods of stress, when the need for elimination is great, while at the same time eliminatory powers are decreased. Man, not even primitive man, has the eliminatory powers of the lower animals. To an increased size of brain and extremities have been sacrificed hepatic, pulmonary and intestinal possibilities. Hence, he needs peculiar care at these periods of stress.

Thorough mastication is desirable, but it is not a panacea. In whatever manner we may eat our food it is desirable, and at periods of stress it is essential, that it be adjusted in quantity to the needs of the body, and nicely proportioned between protein, carbohydrates and fats, for the necessities of the metabolism.

Like all other fads, Fletcherism becomes dangerous, in so far as it is accepted as a cure-all and as a prevent-all. There is almost as much peril in the physical culture nostrums in the hydriatic nostrums, the psychic nostrums, in starving-cures, mastication-cures, and all the other present-day pseudo-medical eccentricities, as there was in the alcohol "bitters" and other almanac-advertised fakes of a generation ago.

People whose digestive apparatus and eliminative organs are going wrong are not good subjects for the medical tinker. They need the careful, individual attention of the best obtainable physicians.—*American Journal of Clinical Medicine.*

*The Open-Air School.* Dr. Leonard P. Ayers, who has done so much to extend the scope and influence of open-air schools, says:

"It is not particularly surprising that children enjoying the hygenic benefits of these schools improve rapidly in their physical conditions, but what has come as a distinct surprise in each school yet opened is that the children, although giving less than half of the regular time to their school studies, do not fall behind in their class standing. On the contrary, the children invariably keep up with their companions who are studying in the ordinary elementary schools and spending about twice as much time on their lessons.

"It must not be forgotten when we are considering expense that a thousand children of school age die each year of tuberculosis in New York City. On the average they have each had about six years of schooling, for which the city has paid about $250.00. This means that a quarter of a million dollars loss each year in the great city is money expended on educating children who die of tuberculosis before growing up. A quarter of a million dollars a year spent in open-air schools designed to prevent this frightful waste would go far toward meeting the entire expense.

"But the matter of expense and preventable money loss are not the most important phases of the problem of the open-air school. Compute as we will we can never arrive at an estimate in dollars and cents of the value of wrecked hopes and ruined homes.

"The open-air school will take its place in the history of education as marking one long step toward that school system of the future in which the child will not have to be either feeble-minded or delinquent or truant or tuberculous in order to enjoy the best and fullest sorts of educational opportunity."—*Hygiene and the Child.*

*The Discovery of the Decade in Connection with Tuberculosis.*

The great discovery of the decade, says Dr. Woods Hutchinson in the *World's Work*, has been the skin tests of Calmett and Von Pirquet. By simply scratching or rubbing a little tuberculin into the skin, a reddening or reaction is produced which enables us to discover the disease at the very earliest stages, when it is as curable as measles and long before it has become infectious to others. With the aid of this test we can now break up each nest of the disease, every focus of infection as fast as we discover it. We can stop our present practice (as illustrated in New York City) of burying 10,000 cases of the disease every year, but breeding 20,000 new ones to take their place, which means that each case before it died infected at least two others.

*The Prevention of Tuberculous Infection in Childhood.*

A writer in *Good Health* (April,1911) calls attention to the details that should be observed in preventing tuberculosis, and points out very properly that young children should not be allowed to play in the sick room of any one who has any disease of the lungs. Playing on the floor of the sick room especially should be absolutely forbidden.

The germs of consumption are more dangerous for children than for adults.

Mothers with tuberculosis should not nurse their infants, as nursing involves a considerable danger to the child and a heavy drain upon the mother's vitality. Mothers should thoroughly wash their hands before preparing the bottles or handling the infant's food.

Patients with pulmonary disease should not kiss any one in the mouth.

Towels, pipes, clothing, handerkerchiefs and other personal articles used by a tuberculosis person should not be used by other members of the family. When consumptives are bedridden their clothing and bedding ought not to be thrown into the common receptacle for soiled clothes. Such things as can be boiled should be boiled as soon as possible, or else soaked for several hours in a disinfecting solution.

*Preventing Colds.* No matter what the weather, says Anne Guilbert Mahon in *Children's Charities*, a baby should never omit his daily constitutional. Nothing so fortifies the system against taking cold as the habit of being much in the open air and of breathing in quantities of the life-giving ozone. No matter what a mother's duties may be she should never neglect this most important one, of giving baby his daily outings.

The bath should, of course, be given daily, the water at the proper temperature—tested by a reliable thermometer. After the bath a baby should be wrapped immediately in a soft, warm blanket and kept covered a much as possible until fully dressed, then a light woolen sacque should be put on over his dress and worn for a while. Of course, he should never be taken out on chilly days until at least an hour has elapsed after the bath.

A frequent cause of colds in babies and in older children, and one which often leads to the growth of adenoids, is the clogging of the nasal passages. After the bath and on coming into the house from outdoors baby's nostrils should be thoroughly cleansed with a small piece of antiseptic cotton on the end of a toothpick. If any discharge or obstruction is noticed the infant's atomizer should be used.

If a mother will watch these details—a baby's bowels and nasal passages, his feeding, baths and clothing and his supply of fresh air—she will be sure to keep him healthy and absolutely free from colds.

Of course, a baby should never be exposed to the contagion of a cold, if possible. He should never be taken near any one suffering from influenza, but, even if he has been exposed to these influences, by accident or necessity, he will be better enabled to withstand the contagion or to throw off the cold quickly if contracted when these details are properly looked after.

*More About Water Drinking With Meals.*

T. B. Hawk, in *The Archives of Internal Medicine,* September 15, 1911, continues his studies on water drinking, this time with reference to the activity of the pancreatic function under the influence of copious and moderate water drinking with meals. As a result of laboratory experiments he concludes that the ingestion of water at meal time, ranging in volume from one-half to one and one-third liters, stimulates the pancreatic function in two ways:

First by direct stimulation of the nervous mechanism of the pancreas, while the water is still in the stomach.

Second, by increased stimulation occurring upon the entrance of the increased volume of acid chyme into the duodenum.

Dr. Hawk says that the drinking of water with meals should bring about a more rapid and complete digestion and absorption of the fat and carbohydrate constituents of the diet, and these observations have been verified by laboratory experimentation.

*The Treatment of Trigeminal Neuralgia.*

Dr. Alfred Fuchs, assistant to the K.-K. Clinic for Psychiatry and Nervous Diseases in Vienna, in an article reprinted from "Einfuehrungen in das Studium der Nerven-Krankheiten fuer Studierende und Aerzte," says:

"In aconitine we have a remedy which has a specific effect in trigeminal neuralgia. To obtain success, two requirements are important. First, the employment of an effective and well-prepared preparation, and, secondly, the following of a system by which the aconitine effect is supplemented by a purgative treatment. It is not easy to obtain an effective preparation. The official tincture of aconite root (German Pharmacopeia) is unreliable. The difference in the effect of the various aconitine preparations evidently depends upon the presence, in variable quantities, of a strongly active aconitine and the less active constituents contained therein. For these reasons the dosimetric pills, made with a solid mass, are more effective. The maximum dose is variable. Under medical administration the quantity given can be properly controlled, since the intoxication symptoms, such as parasthesia of the tongue lips and hands (ulnar region), are indications for the reduction of the dose. A cumulative effect of the drug is not to be feared, because of the catharsis which must be combined with the treatment. The effective ness of proper active catharsis for the relief of pain in neuralgias is widely known, but is unfortunately, little considered in practice. Gussenbauer was the first who emphatically called attention to the importance of active catharsis in the treatment of trigeminal neuralgia.

"Gussenbauer's experience with peripheral resection of the trigeminus branches were so unsatisfactory that he dicontinued this practice almost entirely and enriched the therapy of trigeminal neuralgia with an evidently unessential but really useful detail, so that this clinician's injunction to attend to the bowels has come to be recognized as the most successful method in the treatment of neuralgias."

In other words, "clean out, clean up, and keep clean." And give aconitine. This is supportive of the position which we have maintained for many years: that neuralgia is often, perhaps most often, an effect of intestinal toxemia. Remove this factor and many cases are readily cured.

